# Effects of platelet-rich plasma on corneal re-epithelization and metalloproteinase expression in the cornea of sheep with experimentally-induced infectious keratoconjunctivitis

**DOI:** 10.14202/vetworld.2023.799-810

**Published:** 2023-04-19

**Authors:** Dunia Yisela Trujillo Piso, Mónica Yamile Padilla Barreto, Maria del Pilar Sanchez Bonilla, Analorena Cifuentes Rincón, Omar Leonardo Aristizábal Páez, Carlos Augusto Rengifo, Alexandre Lima de Andrade

**Affiliations:** 1 Department of Veterinary Medicine and Animal Science, Impronta Research Group, Cooperative University of Colombia, Ibagué, Tolima, Colombia; 2 Department of Animal Health, Small Animal Medicine and Surgery Research Group, University of Tolima, Ibagué, Tolima, Colombia; 3 Department of Clinics, Surgery, and Animal Reproduction, College of Veterinary Medicine, Sao Paulo State University, Aracatuba, SP Brazil

**Keywords:** corneal ulcers, experimental model, matrix metalloproteinase, *Moraxella* spp, pinkeye, platelet-rich plasma

## Abstract

**Background and Aim:**

Infectious bovine keratoconjunctivitis is the most crucial ophthalmic disease among ruminants worldwide. *Moraxella* is the bacteria generally associated with this disease and leads to keratitis, conjunctivitis, corneal ulcers, or blindness. Platelet-rich plasma (PRP) effects in corneal ulcers and different ocular superficial diseases in animals and humans are beneficial and enhance rapid healing and improvement, but the effects in infectious keratoconjunctivitis in ruminants are uncertain. This study aimed to examine the effect of PRP on re-epithelization, corneal tissue, clinical signs, and matrix metalloproteinase (MMP) expression in sheep with infectious keratoconjunctivitis.

**Materials and Methods:**

Eighteen sheep were divided into three groups and subjected to a disease-induction experiment. Group 1 (G1) was administered 1.0 mL PRP subconjunctivally, Group 2 (G2) was administered 1.0 mL PRP subconjunctivally and 50 μL gentamicin drops, and the control group (CG) was administered 50 μL saline solution topically every 12 h. Clinical ophthalmologic examination, fluorescein staining, and photography were carried out. Ulcerated areas were measured employing *J-Image* software. Five and eleven days following the procedure, half of the animals from each group were euthanized, and their corneas were evaluated by histopathology and zymography.

**Results:**

Control Group and G2 epithelialized more rapidly. The CG exhibited fewer clinical signs of ocular disease. In histopathological analysis, in G2, alterations were observed only in the epithelium. The CG and G1 exhibited alterations in the epithelium, stroma, and Descemet’s membrane. In zymography, a decline in MMP-2 expression in the animals treated with PRP was detected. Matrix metalloproteinase-9 was significantly expressed in the animals treated with PRP monotherapy, whereas PRP + gentamicin and CG caused a decrease.

**Conclusion:**

Platelet-rich plasma alone did not demonstrate any beneficial effect on re-epithelialization, a decline in clinical signs, tissue alterations, and expression of metalloproteinases. Platelet-rich plasma combined with gentamicin was capable of suppressing MMPs, primarily MMP-9, but do not display positive effects in re-epithelization, reduction of clinical signs, or tissue effects. These outcomes are similar to those discovered in untreated animals, so the use of PRP in patients with infectious keratoconjunctivitis does not offer greater benefits in sheep. Additional research is required to validate the results of PRP use in natural disease presentation.

## Introduction

Pinkeye disease or infectious keratoconjunctivitis is the most crucial disease among ruminants worldwide. In most cases, it stems from the bacterium *Moraxella* and is transmitted through dipteric vectors, namely flies [1]. This bacterium has various virulence factors and generates exotoxins that can change the cornea and conjunctiva. The lesions are characterized as corneal edema, keratitis, and conjunctivitis. In various cases, the lesions can trigger ulcers and corneal vascularization with conjunctivalization [2]. This bacterium is particularly sensitive to antimicrobials, including streptomycin, gentamicin, neomycin, oxacillin, penicillin, sulfonamides, and tetracycline [3]. Conversely, when treating infectious keratoconjunctivitis, therapeutic options other than antibiotics may be required, including those preventing eye perforation, which is why further treatment of corneal ulcers is usually necessary [4]. An overexpression of certain proteinases has been observed in corneal ulcers of domestic animals, primarily matrix metalloproteinases (MMPs). These are a family of zinc and calcium-dependent enzymes, which are categorized according to their substrate specificity into gelatinases, collagenases, stromelysinases, membrane-type metalloproteinases, etc. [5]. Matrix metalloproteinases-2 and MMP-9 are expressed and exhibit activity against altered collagen. In corneal ulcers, these two enzymes have high activity. In contrast, antiproteinase activity is reduced by tissue inhibitors of metalloproteinase (TIMPs), causing rapid degradation of collagen and other components of the extracellular matrix. Therefore, corneal ulcers may be considered a disorder of proteinase homeostasis, which is why suppressing substances should be added to the therapeutic protocol to prevent complications [6].

Some MMP inhibitors include N-acetylcysteine, ethylenediaminetetraacetic acid (EDTA), doxycycline, ilomastat, a-1 proteinase inhibitor, and blood serum, which have shown *in vitro* activity and inhibition of MMP-2 and MMP-9 [6].

Recently, blood products other than blood serum have been added to the therapy for corneal ulcers and including PRP and platelet lysate, among others [7, 8]. Platelet-rich plasma is an autologous concentration of platelets in a small volume of plasma acquired from whole-blood centrifugation. Platelets are rich in growth factors, which are secreted by their alpha granules and are crucial for tissue regeneration since they induce angiogenesis and control inflammation and extracellular matrix deposition [9]. Tanidir *et al*. [10] administered PRP subconjunctivally to rabbits subjected to lamellar keratectomy and observed that it enhanced corneal re-epithelization, which was characterized by a constant migration of fibroblasts, faster epithelial regeneration, and lower inflammation. They reported that the best results were yielded when PRP has applied alone, as opposed to its combination with topical antibiotics. In veterinary ophthalmology, PRP is used and its outcomes may conclude that corneal ulcers in dogs and cats improve rapid re-epithelization [7]. Similar results were achieved by Merlini *et al*. [11] in dogs and in keratoconjunctivitis sicca cases in dogs [12]. To date, the study on the effects of PRP on metalloproteinases is limited and the results are controversial. Perches *et al*. [13], in research regarding corneal ulcers treated with PRP, demonstrated that the use of eye drops of platelet-rich and poor plasma affects the expression of matrix metalloproteinases participating in the corneal repair process.

Farghali *et al*. [7] administered PRP to dogs and cats with corneal ulcers of diverse etiology and concluded that there was a significant decrease in MMP-2 and MMP-9 expression when compared with a control, as concluded from zymography. They reported that the outcomes were clinically significant since the animals exhibited full healing and re-epithelization of their ulcers within approximately 2 weeks, alongside recovery of corneal transparency. Contrarily, Sakimoto, *et al*. [14] validated that PRP administration to patients with recurrent corneal erosion improved the MMP levels when the platelet concentrations increased. Conversely, it should also be noted that the levels of their inhibitors (TIMPs) increase when the MMP levels elevate. Similar results were established by Pifer *et al*. [15] because they inferred that PRP increased the MMPs expression and it’s dependent on the leucocytes quantity.

This study aimed to examine the clinical effects, histological effects, and the MMPs expression of PRP on corneal ulcers infected with *Moraxella ovis*. This is so as to recognize the properties of PRP and if its use is beneficial in the treatment of corneal ulcers infected with *Moraxella* spp. in sheep.

## Materials and Methods

### Ethical approval

This research involves animals and its ethical management was approved by the Ethics Committee of the Universidad Cooperativa de Colombia under the file and number AEC 003/2018 and 015-2018.

Details and information about animals’ ethic management were considered, following the suggestion by the ethics committee that included an ophthalmic assessment format for each member that participated in the study and who analyzed the severity of the lessons. Extended informed consent was signed by the responsible person regarding the animals, a treaty agreement from researchers with the ethics committee, patients healthiness certificate provided by the ovine supplier, and were established the final judgments. Contrarily and aimed to fulfill Russel and Burch’s postulates regarding replacement, refinement, and reduction (3Rs), the study included six animals in each study group, managed in independent farmyards as Gould *et al*. [16] recommended, preventing nasal contact between patients and besides achieving with optimal conditions on animal welfare but modified to the proposed experimental model.

Animal monitoring evaluation was developed with three daily inspections, which were included in addition to the examination of each individual in two of them and flunixin’s meglumine application by intramuscular injection (2.2 mg/kg) SID (once daily), during 5 days aimed at reducing pain and swelling present after surgery. It is important to consider that for making corneal ulcers, which is considered the most painful treatment in patients, pre-anesthetic medication was performed with xylazine, whose value in reducing the pain is crucial in these cases, and deep general anesthesia in trans–surgical periods.

No significant signs of extreme pain were observed in the patients at any time of the study, characterized by discomfort or others; contrarily, they preserved their intake and activity in ideal conditions, without variations worthy of mention or exclusion from the experiment.

### Study period and location

The study was conducted from January 2019 to November 2020 at Faculty of Veterinary Medicine and Animal Sciences, Ibagué, Tolima - Colombia. The procedures involved for *Moraxella* spp. were performed under the biosafety and biosecurity regulations.

### Study animals

The research included 18 clinically healthy female adult sheep of the Colombian Criollo breed.

The study design included three study groups with six sheep each. All animals were subjected to axial lamellar keratectomy of the right eye, followed by the immediate inoculation of a strain of *Moraxella ovis* in the cornea.

The first group (G1) was administered 1.0 mL of PRP subconjunctivally. The second group (G2) was administered 1.0 mL PRP subconjunctivally and topical therapy with antibiotics consisting of 50 μL of gentamicin three times a day. The Control Group (CG) was administered topical therapy with 50 μL of saline solution three times a day. All animals received 2.2 mg/kg flunixin meglumine every 24 hours intramuscularly for pain and inflammation management over a period of 5 days. Treatment was initiated 24 hours after corneal ulcer induction and bacterial inoculation (except for non-steroidal anti-inflammatory drugs).

The animals were subjected to general clinical and ophthalmology analysis and procedures such as corneal ulceration, bacterial inoculation, PRP treatments, and others were made. They were enclosed in individual pens with mosquito netting under strict isolation parameters and hygiene conditions and were given a diet according to fodder crops, concentrated food, and water. In addition, one cornea from a sheep slaughtered in the Ibagué meat packing plant was included for use as basal in statistical comparisons of zymography. The sheep was a healthy animal and did not have a clinical or ophthalmic disease. This cornea was known as a “healthy cornea.”

### Corneal ulceration

Before anesthesia, the animals received a dose of 0.1 mg/kg of xylazine (Rompun^®^; Bayer, Leverkusen, Alemania) intravenously; propofol (4 mg/kg) was employed for induction, and anesthesia was preserved with 0.05 mg/kg/min propofol infusion.

**Figure-1:**
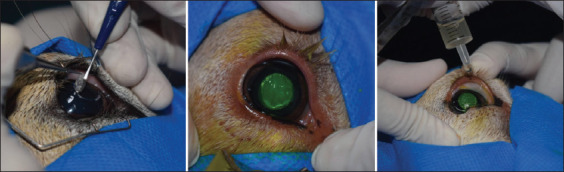
Experimental model of infectious keratoconjunctivitis where the making of the corneal ulcer can be observed, its visualization through the fluorescein test and the inoculation of the bacterial strain.

The animals were placed in the left lateral decubitus, and a periocular trichotomy was carried out. Drops of local anesthesia were administered employing topical proxymetacaine, and antisepsis of the cornea and conjunctiva was attained with polyvinylpyrrolidone iodine diluted in physiological saline solution (1:50). The eye was centralized with Nylon 6-0 suture employing scleral-conjunctival guiding sutures. Axial lamellar keratectomy was conducted on the right eye with an 8-mm punch and a 2.0 mm crescent scalpel angled (Figure-1).

### Microorganisms and inoculation

A pure rural *M. ovis* strain was acquired from the culture of conjunctival swabs obtained from sheep with clinical keratoconjunctivitis. The strain was isolated in 5% sheep blood agar (with tryptic soy agar base) (BD BBL™ Sparks, USA) at 37°C for 20 h, followed by suspension in a sterile saline solution. The suspension was adjusted to 1 × 10^9^ colony forming units (CFU)/mL, which corresponded to an optical density at 600 nm of 0.4–0.6 (McFarland scale). The suspension was stored at 4°C to prevent bacterial growth and, after less than an hour, 0.1 mL was inoculated in the eye in which the superficial lamellar keratectomy was carried out.

Inoculation was verified by sampling with conjunctival swabs on days 3 and 11 after the inoculation. The samples obtained with the conjunctival swabs were seeded on lamb’s blood agar (with soybean trypticase agar base) (BD BBLTM Sparks, USA) for 20 h at 37°C. Biochemical and morphological tests such as Gram, observation of coccobacilli forming chains or in pairs, oxidase, catalase, urease, and carbohydrate fermentation were developed to determine the bacteria that grew on the agar.

### Platelet-rich plasma

For each sheep in Group 1 (G1) and Group 2 (G2), 5 mL of whole-blood was obtained in a citrate tube by jugular venous puncture. 1 mL of the blood was used for blood count and manual platelet count. The remaining blood was centrifuged for 5 min at 224× *g* at a temperature of 21°C–24°C to separate the blood components. A Pasteur pipette was employed to collect 1 mL of the substance located between the lower plasma portion and the upper erythrocyte portion, which was transferred to a tube for double centrifugation. In this way, we got the target volume of 1 mL PRP. From this tube, 100 μL were collected and placed in a Neubauer chamber for the platelet count to guarantee adequate platelet concentration.

### Measurement of ulcerated areas

**Figure-2:**
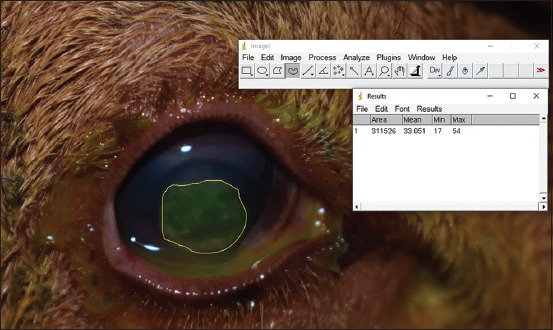
Measurement of the corneal ulcerated area using Software Image J. The free-hand tool delimited the area in each moment of evaluation.

The ulcerated corneas were examined by slit lamp biomicroscopy for the tabulation of clinical signs, and visualization with a cobalt blue filter was done after conducting the fluorescein test every 12 h by the same observer immediately following keratectomy and until the test was negative. The eyes were photographed employing a D3300 (Nikon, Tokyo, Japan) camera at a fixed focal length of 12 cm. The images were evaluated using Software Image J (NIH, Bethesda, USA), which measured the ulcerated area and its evolution. The ulcerated area was delimited using the free-hand selection tool and values were collected (Figure-2).

### Cornea collection

Five days following the study procedure, half of the animals in each group were sedated with 0.2 mg/kg intramuscular xylazine and then euthanized with pentobarbital sodium (Euthanex^®^, Invet, Bogotá, Colombia) 100 mg/kg administered intravenously. Their eyes were enucleated and their corneas were dissected to evaluate the corneal tissue. The same procedure was conducted on the remaining animals on day 11. Corneas were split in half and one of them was preserved in formaldehyde, while the rest was frozen and collected in an Eppendorf tube and preserved at −70°C for zymography.

### Zymography

To evaluate the proteolytic gelatinase-type activity of MMPs in the cornea, proteins were extracted from portions of the sheep cornea. First, 50 mg of the tissue was cut, weighed, placed in 1 mL of radioimmunoprecipitation assay, and polytron cold homogenization was carried out for 60 s. After maceration, the preparation was incubated for 20 min, maintaining its low temperature, followed by cold centrifugation (4°C) 20160× *g* for 20 min. The supernatant was recovered for gelatinase activity analysis. Proteins were measured.

The protein samples (20 mg) were applied to 10% polyacrylamide gels copolymerized with 0.2% porcine skin gelatin (Sigma–Aldrich, Merck, Darmstadt, Germany) to collect the substrate to be degraded and subjected to 150V electrophoresis (Thermo-Fischer Scientific, Waltham, USA) at 4°C. Next, the gels were washed 5 times, twice with 4% Triton X-100 for 20 min each and thrice with distilled water for 5 min each at room temperature (27°C) with constant agitation. After washing, the gels were incubated in zymography buffer for 60 min at 27°C, and then incubated at 37°C in the same type of buffer with constant agitation for 18 h. The gels were stained with Coomassie Blue G-250 (Thermo-Fischer Scientific) and destained with water to see the clear bands showing gelatinase activity. The gels were recorded on Image Lab™ software (https://www.bio-rad.com/es-co/product/image-lab-software?ID=KRE6P5E8Z). Densitometric analysis was carried out employing GelAnalyzer (GelAnalyzer 19.1, www.gelanalyzer.com) which automatically detects the existing lanes. The background was eliminated from each lane by the rolling ball method. Considering the intensity of all the pixels in each lane, the bands corresponding to the highest intensity areas were identified. This software yielded a numerical value named raw volume, which was employed for comparative analysis.

### Histopathology

For histopathological analysis, cornea samples preserved in formalin were added to liquid paraffin and once the blocks were produced, cuts were made with microtome; these were subsequently fixed on slides and stained with Hematoxylin-eosin. The plates were observed in an electron microscope Leica (Leica microsystems, Wetzlar, Germany) utilizing 10, 40, and ×100 objectives of magnification.

### Statistical analysis

To examine the behavior of the ulcerated area-surface in the study groups at each sampling moment to identify the groups with a more efficient recovery from the corneal lesion, an assessment of variance was employed with a confidence level of 95%, followed by Tukey’s multiple comparison tests.

The presence or absence of each clinical sign and histopathological finding in the groups several times was compared employing the Chi-square test with a significance of 5%. This was performed to identify any clinical signs that occurred more frequently in one of the study groups during the experiment.

The data received by zymography as densitometric units were tabulated and evaluated for normality utilizing the Shapiro–Walk test. Each sampling point was analyzed by analysis of variance with a 95% confidence level; a Tukey’s test was then performed to compare groups and identify the differences.

## Results

### Platelet-rich plasma

The standardization technique employed to collect PRP permitted a 2.45-fold concentration of the value observed in the blood of each animal in G1 and G2.

### Analysis of ulcerated surfaces

At all assessment times, there was a reduction in ulcerated areas, most remarkable in CG. Thirty-six hours after the injury, the group of animals treated with PRP + gentamicin had the largest ulcerated area, which was significantly different from that of CG (p = 0.019) but was not significantly different from that of G1.

When comparing the ulcerated area of the study groups at 60 and 72 h, CG had the smallest diameter, which varied from G2, which had a greater ulcerated surface area similar to the assessment at 36 h. Group 1 did not significantly differ from CG and G2. Then, at 84, 96, and 108 h, a statistical difference was found between CG, G1, and G2, where CG had less ulcerated area. There was no significant difference in the other examination moments.

**Figure-3:**
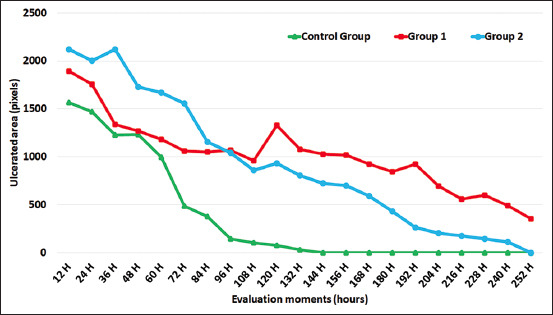
Mean of the ulcerated surface of each group at each of the evaluation moments. Of the treated animals, those that received platelet-rich plasma + gentamicin showed more rapid re-epithelialization. The control group re-epithelialized the cornea in a shorter time.

**Figure-4:**
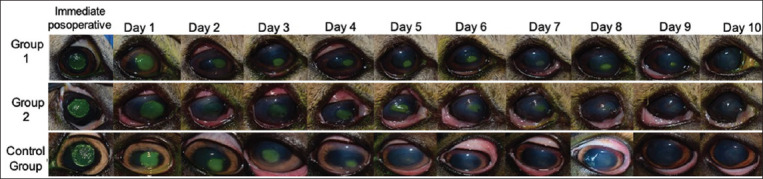
Follow-up photographic sequence of the ulcerated corneal area of one patient from each study group. Fluorescein test.

In corneal recovery assessment (re-epithelization time), CG demonstrated a faster recovery of ulcerated areas, with fewer ulcerated surfaces than the other groups at all monitoring times (p = 0000). Group 2 exhibited a greater recovery than G1. There was statistical proof to verify that the animals in CG had a faster corneal epithelium recovery than those in G1 and G2 (Figures-3 and 4).

### Specific ocular clinical signs

Twelve clinical signs were observed in the analyzed animals: Blepharospasm, ciliary injection, conjunctival hyperemia, corneal conjunctivalization, corneal edema, corneal stromal abscess, corneal vascularization, epiphora, mucus discharge, photophobia, purulent discharge, and serous discharge.

On day 1 of the experiment, 83.3% of the animals in G1 and 100% in G2 presented with epiphora, which was absent in CG, so there was a significant difference between CG and G1 and 2 (p = 0.0007). All the animals in G1 and G2 had blepharospasm, which was not present in CG; therefore, there was a significant difference between CG and the remaining study groups (p = 0.0001). All animals in G1 and 50% of the animals in G2 had corneal edema, which was significantly different in CG in which no study subject exhibited this clinical sign (p = 0.0025).

At the second time of monitoring, all patients in G1 and G2 presented with corneal edema, which was only found in 50% of the animals in CG, finding a statistical difference between CG and G1 and 2 (p = 0.0273).

Conjunctival hyperemia was more frequent in G1 (83.3%), which was significantly different (p = 0.0139) than that in G2 in which only 50% of the patients were impacted, and CG in which no animal exhibited this alteration. The same frequency was seen with ciliary injection (p = 0.0498).

On the 3rd day of the experiment, conjunctival hyperemia and serous secretion were absent in CG, so it was significantly different compared to G1 and G2 (p = 0.0018 and p = 0.0007, respectively). Serous secretion was observed in 100% of the animals in G2. Unlike these clinical signs, blepharospasm only appeared in 50% of the animals in CG (p = 0.0273).

On day 4, epiphora and blepharospasm were the only clinical signs that exhibited significant differences between the study groups. Only 16.7% of the patients in CG had epiphora compared to 83.3% in G1 and G2 (p = 0.0237). No patient in G1 and G2 had blepharospasm, but 50% of the patients in CG had it (p = 0.0273).

**Figure-5:**
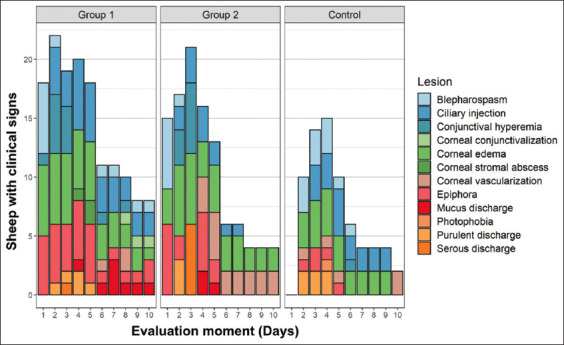
Ophthalmic clinical signs observed daily in sheep with experimentally-induced infectious keratoconjunctivitis. Group 1: Platelet-rich plasma, Group 2: Platelet-rich plasma + Gentamicin, Control group: Saline solution.

On day 5, corneal vascularization between the study groups was significantly different (p = 0.0273). It was observed in 66.7% of the animals in G2, then 16.7% in CG, and none in G1. Between day 6 and day 10 of the evaluation, there were statistically significant differences between the clinical signs detected (p > 0.05) (Figure-5).

### Histopathological findings

Histopathological findings on day 5 showed 13 histological alteration types (Table-1).

**Table-1:** Histopathological findings in sheep patients with induced infectious keratoconjunctivitis after 5 days.

Histopathological findings: 5 days	Group			p-value	Corneal layer

	Control	Group 1	Group 2		
	No. of patients	No. of patients	No. of patients		
Increased cellularity in Bowman’s membrane	2	0	1	0.2231	Epithelium
Descemet membrane thickening	1	0	0	0.3247	Descemet membrane
Intracellular edema of the anterior corneal epithelium	1	0	1	0.5258	Epithelium
Corneal vascularization	0	1	2	0.2231	Epithelium
Estromal edema	1	0	0	0.3247	Stroma
Segmental atrophy of the anterior corneal epithelium	1	0	0	0.3247	Epithelium
Severe ulcer	0	0	3	0.0111	Epithelium
Inflammatory infiltrate in corneal epithelium	0	2	0	0.0764	Epithelium
Thinning of the anterior corneal epithelium with prominent stratum lucid	0	1	0	0.0764	Epithelium
Intracellular edema of basal cells	1	1	0	0.5258	Epithelium
Marginal irregularity in descemet membrane	0	1	0	0.3247	Descemet membrane
Severe thinning of corneal epithelium	0	1	0	0.3247	Epithelium
Stromal focal purulent inflammation	0	1	0	0.3247	Stroma

**Figure-6:**
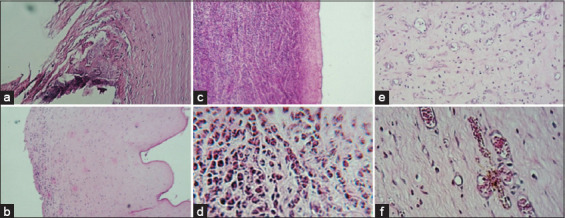
Photographs of the main corneal histological alterations found at evaluation moment 1 (Day 5). In (a and b) severe corneal ulcer, notable loss of corneal epithelium and stromal exposure. In (c and d) inflammatory infiltrate with a predominance of neutrophils, in (e and f) corneal vascularization.

In G1, CG, and G2, 53%, 46%, and 31% of these were observed, respectively. Once the histological alterations were characterized, they were categorized based on the affected corneal layer: Epithelium, stroma, and Descemet’s membrane. The alterations at this time of assessment were primarily seen in the corneal epithelium in the three assessment groups. It should be noted that in G2, Descemet’s stromal and membrane lesions were not observed, while these were observed in the other study groups. When comparing the histopathological findings in each study group, a statistical difference was observed only in tissue alterations characterized as severe ulcers, which was more frequently seen in G2 compared to CG, where the injury was not reported (Figure-6).

A total of 15 histopathological results were observed in the second moment of corneal tissue evaluation (Table-2).

**Table-2:** Histopathological findings in sheep patients with induced infectious keratoconjunctivitis. Day 5 after experimental induction.

Histopathological findings: 11 days	Group			p-value	Corneal layer

	Control Group	Group 1	Group 2		
		
	No. of Patients	No. of Patients	No. of Patients		
Bowman’s membrane fibrosis	0	2	3	0.0429	Epithelium
Basal membrane thickening in corneal epithelium	0	0	1	0.3247	Epithelium
Hemidesmosome lysis	2	0	0	0.0764	Epithelium
Intracellular edema of the anterior corneal epithelium	0	1	1	0.5258	Epithelium
Keratinization	1	0	1	0.5258	Epithelium
*Rete ridges*	2	0	1	0.2231	Epithelium
Recurrent superficial injury	1	0	0	0.3247	Epithelium
Increased thickness of Bowman’s membrane	1	0	0	0.3247	Epithelium
Stromal vascularization	1	1	1	1.0000	Stroma
Stromal edema	1	0	0	0.3247	Stroma
Stromal hyperplasia	1	0	0	0.3247	Stroma
Stromal inflammatory infiltrate	0	2	2	0.1653	Stroma
Epithelial ulcer	0	1	0	0.3247	Epithelium
Thinning of the anterior corneal epithelium	0	0	1	0.3247	Epithelium
Epithelial vascularization	0	1	0	0.3247	Epithelium

**Figure-7:**
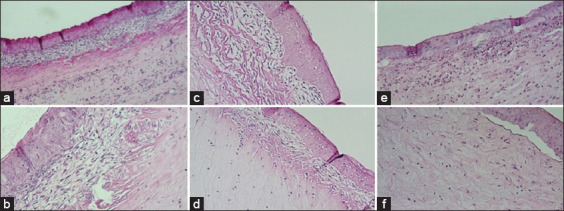
Photographs of the main histological alterations found in the corneas of the sheep at time 2 (Day 11). In (a and b) Bowman’s membrane fibrosis, in (c and d) rete ridges, in (e and f) hemidesmosome lysis.

In CG and G2, 53% of these were seen, while it was only observed in 50% of the animals in G1. The observed alterations were majorly found in the epithelium, followed by the stroma. Descemet’s membrane involvement was not found in any groups. G1 had the least number of alterations. Bowman’s membrane fibrosis alteration was discovered in G2, which was significantly different in comparison with CG but was not significantly different in G1. A total of three stromal alterations and an average of seven epithelial abnormalities were found in each study group. *Rete ridges* and lysis of hemidesmosomes happened in CG in crucial frequency but without statistically significant difference (Figure-7).

### Zymography

**Figure-8:**
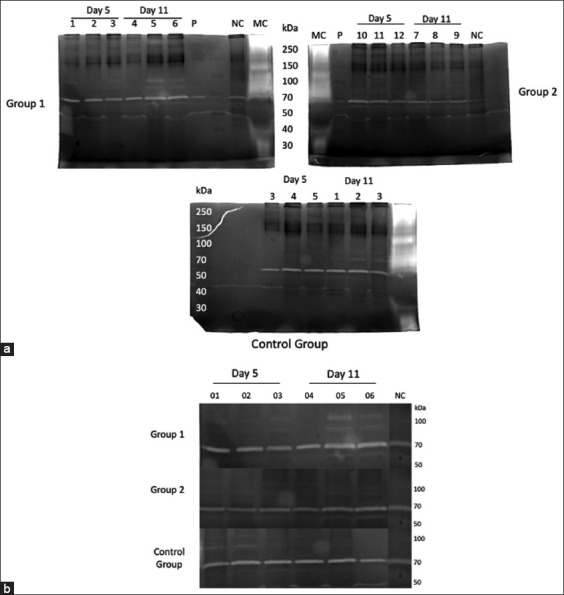
Original (a) zymography gels and cuts (b) showing matrix metalloproteinase (MMP)-2 (72 kDa) and MMP-9 (92 kDa) activity in the three study groups: Group 1 treated with platelet-rich plasma (PRP), Group 2 treated with PRP + gentamicin, and Control Group.

Latent and active MMP-2 were seen in the corneas of the study animals alongside the healthy animals (healthy cornea, HC). Active MMP-9 was observed in Group 1 (subconjunctival PRP alone), G2, and CG (subconjunctival PRP + gentamicin TID) (Figure-8).

### Active MMP-2

Based on active MMP-2 polyacrylamide gel analysis, the bands with the highest intensity were in the CG, followed by G2. When evaluating active MMP-2 expression on days 5 and 11, there was a significant difference (p ≤ 0.05) among all study groups and healthy corneas, producing higher expression values for ulcerated corneas.

When conducting a variance analysis, a statistically significant difference was detected among the groups on day 5 (p = 0.0001). In the multiple comparison tests, it was discovered that the CG was the one with the highest MMP-2 expression, with a statistically significant difference from the other groups. There were no significant differences between Groups 1 and 2. The healthy cornea demonstrated lower MMP-2 expression, with a significant difference from the CG and Group 1; still, no differences from G2 were observed.

Also, on day 11, a statistically significant difference was found among the groups, with p = 0.002. Again, the CG showed higher MMP-2 expression, with significant differences from G1 and the healthy cornea, but with no differences from G2. There was no statistical differences between Groups 1 and 2.

**Figure-9:**
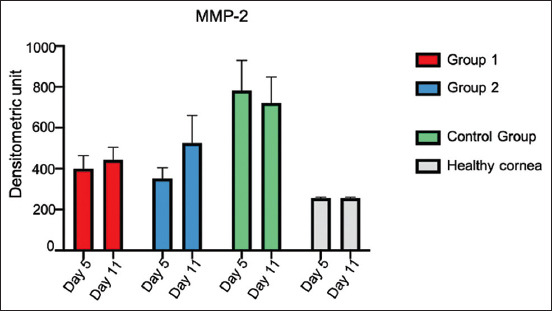
Densitometric values obtained from the quantification of latent matrix metalloproteinase-2 expression in the cornea of sheep subjected to an experimental model of infectious keratoconjunctivitis and treated with platelet-rich plasma alone and combined with gentamicin on days 5 and 11 after lamellar keratectomy and bacterial inoculation.

The healthy cornea had lower MMP-2 expression, with statistical differences from G2 and the CG, but with no differences from G1 (Figure-9). When assessing the MMP-2 expression in all groups and at both testing points, higher expression was observed in the CG on comparing the healthy cornea with the remaining study groups.

### Matrix metalloproteinase-9

Comparisons were formed between study groups (G1, G2, and CG) and between study groups and healthy corneas.

The analysis of the polyacrylamide gel and the densitometric analysis of latent MMP-9 demonstrated the expression of this metalloproteinase, particularly in the group treated with PRP alone (G1). Matrix metalloproteinase-9 expression was very low in the CG and the group treated with PRP+ gentamicin (G2).

When evaluating MMP-9 expression at the first testing point, a clear statistical difference (p = 0.0250) was observed between G1 and the healthy cornea. Matrix metalloproteinase-9 expression was high in G1. When comparing MMP-9 expression between G1, G2, and the CG, no differences were detected.

**Figure-10:**
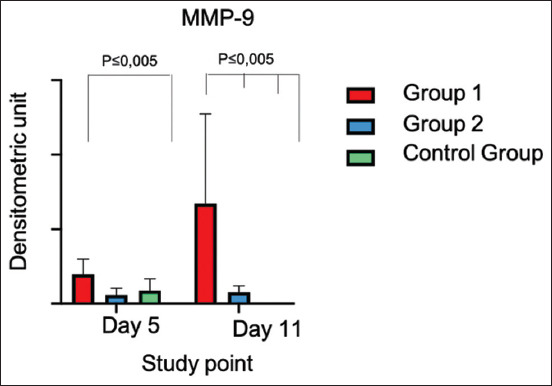
Matrix metalloproteinase-9 expression in the cornea of sheep with experimentally-induced infectious keratoconjunctivitis treated with platelet-rich plasma alone (Group 1) and platelet-rich plasma combined with gentamicin (Group 2) as opposed to the Control Group at two testing points.

At the second testing point, in which zymography was conducted, there was a statistically significant difference between G1 and the remaining groups (0.0030). Group 1 still exhibited the highest MMP-9 expression values, followed by G2. No MMP-9 expression was detected for the CG and the normal cornea (Figure-10). When comparing the MMP-9 expression of all the study groups and at both testing points, higher expression was observed in Group 1.

## Discussion

In our research, the saline-treated group (CG) was the group with the best outcomes regarding ulcer area reduction, assessed in time and lesion size. These animals re-epithelialized more rapidly than the groups treated with PRP and the diameter of the ulcers was lowered more efficiently. These differences cause us to imply that PRP does not aid the repair process of corneal ulcers and that, contrarily, its use generates delays in corneal healing, similar to what was previously reported by Anitua *et al*. [17].

These outcomes may also be because PRP was applied 24 h after starting the experiment since it was the required time to replicate the infectious keratoconjunctivitis model. Nevertheless, this period could have functioned against re-epithelialization, since an early injection of PRP (2 h) in patients with corneal ulcers enhances an efficient restoration of the corneal epithelium, unlike in patients who received it at a later time [18].

After 36 h, G2 demonstrated a reduction in ulcer diameter, thus evidencing a better and more efficient corneal recovery compared with G1. Antibiotics could have boosted the re-epithelialization response because of the susceptibility of *M. ovis* to gentamicin [19]. These outcomes verified the theory of Tanidir *et al*. [10], considering that prophylactic antibiotics are required in corneas vulnerable to infections during healing to prevent complications.

The antimicrobial effect of PRP is not well-known, but recent research considers that its use can suppress bacterial growth and therefore contribute more sufficiently to re-epithelialization [20]. In this research, a monotherapy with PRP for corneal ulcers contaminated with *M. ovis* induced a slower epithelialization compared to treatment with both PRP and antibiotics and untreated animals, which implies that in the presence of bacteria, such as *M. ovis*, PRP only is inefficient.

Poor results of PRP in our research may be linked to the application of a single dose of this blood product because some researchers recommend that at least two doses of PRP are needed to yield positive results in terms of tissue repair [18]. Regardless, some postulates suggest that at least two doses of PRP are needed to attain positive results in terms of tissue repair [7]. In this research, only one application of PRP was made based on the results of prior research, and considering the need to prevent undesirable situations in the restraint of patients, it is inevitable to recommend that a single dose of PRP did not exhibit optimal results regarding recovery time, since when the results obtained in CG are compared with those in groups treated with PRP, the first one presented with a faster re-epithelialization speed, and all the animals treated with PRP had a lowered ulcerated corneal area in a lower speed. The CG showed the least amount of clinical alterations similar to what was observed in the evaluation of the ulcerated area, although signs of pain were the most observed constant throughout the assessment time. Various researches affirm that PRP helps ease the pain progressively [21]. The positive results in pain reduction are associated with growth factors, primarily transforming growth factor-β, which has a significant anti-inflammatory role, and the inhibition of interleukin-1 (IL-1) and MMP-3 and cyclooxygenase (COX-2) reduction [22].

The positive results observed in the CG can be linked to only be similar to those obtained in the group treated with PRP + gentamicin. Nevertheless, the outcomes in these two groups were far from the results observed in the group treated only with PRP. This implied that in sheep with infectious keratoconjunctivitis, PRP should be administered concomitantly with an antibiotic, but their application as monotherapy is not recommended.

Based on the clinical signs, corneal edema was observed with high frequency. Corneal edema in ulcerative keratitis occurs because once the hydrophobic corneal epithelium is lost, the highly hydrophilic stroma is exposed. Some studies recommend that corneal edema is also a reflection of leukocyte infiltration, implying that PRP induced the corneal edema because the number of white blood cells in PRP was assumed to be high. Some studies suggest that leukocyte-PRP (L-PRP) drastically lowers vascular endothelial growth factor (VEGF) values and elevates the levels of pro-inflammatory cytokines, such as IL-1B and IL-16, forming a more pro-inflammatory environment [23].

The invasion of blood vessels or ciliary injection is also frequently observed in corneal ulcers, mainly in those with extensive lesions. This vascularization lowers corneal transparency because there are also pigment deposition and inflammatory cells. This outcome was observed in all assessment times and all patients treated with PRP; this reveals the extensive ulcerated areas generated by the lack of antibiotics and by the presence of VEGF and platelet-derived growth factor in PRP that trigger angiogenesis [24].

The group treated only with PRP presented with minor histologic alterations compared to the other groups, which may indicate the epitheliotoxicity of gentamicin, as demonstrated by Fernández-Ferreiro *et al*. [25]. The choice of gentamicin was taken into account due to its routine application in sheep and goat productions in the region, but its outcomes indicate the need for other alternatives that lack this toxicity.

To date, the effects of PRP on metalloproteinases have been scarcely examined and the results are controversial. Perches *et al*. [13], in research regarding corneal ulcers treated with PRP, demonstrated that the use of eye drops of platelet-rich and poor plasma affects the expression of matrix metalloproteinases involved in the corneal repair process.

Farghali *et al*. [7] administered PRP to dogs and cats with corneal ulcers of diverse etiology and concluded that there was a significant reduction of MMP-2 and MMP-9 expression if compared with a control, as inferred from zymography. They reported that the outcomes were clinically significant since the animals exhibited full healing and re-epithelization of their ulcers within approximately 2 weeks as well as recovery of corneal transparency.

Contrarily, Sakimoto *et al*. [14] evidenced that PRP administration to patients with recurrent corneal erosion improved the MMP levels when the platelet concentrations increased. Nevertheless, it should also be noted that when the MMP levels increase, the levels of their inhibitors (TIMPs) also increase. Similar results were observed by Pifer *et al*. [15] because they concluded that PRP increased the MMPs expression and it’s dependent on the leucocytes quantity. Neither PRP applied as a single treatment nor PRP applied in combination with gentamicin was capable of totally inhibiting metalloproteinase expression at the two study points.

Our outcomes indicate that PRP decreases the expression of MMP-2, but increases the expression of MMP-9. Matrix metalloproteinase-9 overexpression leads to extensive damage to the cornea, namely, the destruction of cell adhesion structures in the epithelial cells and delayed re-epithelization of the ulcerated cornea [26].

Poor response of the PRP-treated cornea regarding MMP-9 suppression may be linked to the high leukocyte counts in the obtained plasma, as recommended by Pifer *et al*. [15], who identified the number of leukocytes in PRP (L-PRP) and showed that MMP expression is proportional to the leukocyte counts. Leukocytes were not counted in this study, but we suspect that their levels must have been high since we had to use various methodologies previously proposed for other species to acquire sheep PRP with sufficient platelet concentration. Once the platelet concentration procedure was standardized, we concluded that to obtain PRP with 2- or 3-fold concentration levels, we had to extract the portion that was most closely associated with leukocytes (buffy coat); thus, their quantification should be considered in the future research.

Since MMP-9 expression was lower when using PRP combined with gentamicin than when applying PRP alone, the benefits of gentamicin should be taken into account. This combination is necessary, and some studies have also recommended using a local antibiotic release system together with PRP (mixed), which guarantees bacterial elimination and accelerates regeneration [27].

The outcomes from CG implied a significant increase in MMP-2 expression and a minor expression in patients treated with PRP. It may reveal that the PRP is can reduce the MMP-2, principally if PRP is combined with gentamicin because the MMP-2 expression is similar to the healthy cornea.

The lowest level of MMP-9 expression was observed in the CG, which was barely noticeable on day 11. Hence, we could conclude that PRP administered alone or in combination does not lower MMP expression, particularly in the case of MMP-9. Untreated sheep with infectious keratoconjunctivitis or sheep subjected to hydration of the cornea and reduced manipulation with non-invasive drug administration, such as topically rather than subconjunctivally, are likely to exhibit reduced inflammation and consequently lower MMP production, causing a more efficient repair process [28].

## Conclusion

In ovine species and corneal diseases of these, namely, infectious keratoconjunctivitis, PRP is ineffective if applied as monotherapy, because it is inefficient in lowering the size of corneal ulcers. It does not decrease the clinical signs of the disease and increases the expression of MMPs. The poor effectiveness is due to the PRP extraction methods, which in this species have certain peculiarities, particularly because for its extraction, following centrifugation, the platelets descend and are located extremely close to the white blood cells and it inevitably obtaining a plasma rich in platelets and leukocytes too, the latter being large producers of MMPs.

Platelet-rich plasma combined with antibiotic therapy may suppress MMP-2 and a marked MMP-9. However, these findings are similar to those revealed in untreated animals, so the use of PRP with or without gentamicin in patients with infectious keratoconjunctivitis does not deliver greater benefits in sheep.

The leukocytes in the PRP must be measured to ascertain the correlation between a high number of these cells and the poor outcomes in corneal re-epithelialization and the overexpression of metalloproteinases; all this because the platelet concentration techniques were only possible if platelets were collected close to the buffy coat.

## Authors’ Contributions

DYTP, ALA, and OLAP: Design the study, material preparation, and data collection DYTP, MYPB, MPSB, and CAR: Supervision and project administration. DYTP, MYPB, and ACR: Data analysis and revised the manuscript. DYTP and ALA: Drafted the manuscript. All authors have read, reviewed, and approved the final manuscript.
